# How Safety Climate Impacts Safety Voice—Investigating the Mediating Role of Psychological Safety from a Social Cognitive Perspective

**DOI:** 10.3390/ijerph191911867

**Published:** 2022-09-20

**Authors:** Yunfeng Sun, Hao Yang, Xiang Wu, Yifeng Jiang, Chongyang Qian

**Affiliations:** 1 School of Engineering and Technology, China University of Geosciences (Beijing), Beijing 100083, China; 2 China Electric Power Research Institute, Beijing 100192, China; 3 Institute of Urban Safety and Environmental Science, Beijing Academy of Science and Technology, Beijing 100054, China

**Keywords:** safety voice, safety climate, psychological safety, mediating role, “environment-subject cognition-behavior” triadic interaction model

## Abstract

Safety voice has become a popular research topic in the organizational safety field because it helps to prevent accidents. A good safety climate and psychological safety can motivate employees to actively express their ideas about safety, but the specific mechanisms of safety climate and psychological safety, on safety voice, are not yet clear. Based on the “environment-subject cognition-behavior” triadic interaction model of social cognitive theory, this paper explores the relationship between safety climate and safety voice, and the mediating role of psychological safety. We collected questionnaires and conducted data analysis of the valid questionnaires using analytical methods such as hierarchical regression, stepwise regression, and the bootstrap sampling method. We found that safety climate significantly and positively influenced safety voice, and psychological safety played a mediating role between safety climate and safety voice, which strengthened the positive relationship between them. From the research results, it was clear that to stimulate employees to express safety voice behavior, organizations should strive to create a good safety climate and pay attention to building employees’ psychological safety. The findings of this paper provide useful insights for the management of employee safety voice behavior in enterprises.

## 1. Introduction

There are numerous production accidents in today’s society, where the scale of enterprises is growing. Measures have been taken in terms of legal norms, management standards and systems, and the safety environment of enterprise production has been significantly improved. However, the current safety situation is still in an alarming state, in particular, the frequency of serious accidents and serious hazards. Safety accidents are harmful to organizations, so taking effective measures to reduce the accident rate is the top priority for an enterprise’s development [1]. Safety voice is considered to be the antecedent that helps avoid accidents [2,3,4], and the importance of safety voice in organizations is becoming more and more prominent. However, research shows that most employees will choose silence instead of safety voice, because they are worried about the adverse consequences of safety voice behavior [5]. Therefore, how to stimulate safety voice is important, to prevent enterprise production accidents and optimize safety management systems.

Research shows that safety climate affects safety-related behaviors [6,7,8,9]. Safety voice is a challenging behavior. When employees express safety voice, they often require the organization to change their rules and procedures [10], which may cause trouble for colleagues, exposing employees to face a greater interpersonal crisis. In addition, the indifferent attitudes of leaders toward safety issues can inhibit employees’ willingness to talk about safety matters [11]. Safety climate reflects employees’ understanding of the priority of organizational safety. Employees form their judgments based on corporate systems, practices, and procedures, and, thus, exhibit different attitudes and behaviors toward safety [7,8,12]. An organization’s priority for safety is judged by its safety climate [13]. When the priority for safe work is high, the organization allocates more resources to safety. When employees feel that safety is taken very seriously, they will be less likely to consider the risks of safety voice. Whether or not employees dare to express safety voice behavior is influenced by the safety climate, and a good safety climate tends to motivate employees to express their ideas about safety [7,14,15]. Some studies have revealed the positive role of safety climate on employee voice [16,17]. However, there are differences between safety voice and employee voice, for example, severity [18]. Therefore, how safety climate works for safety voice needs particular discussion. The study of safety behavior is increasingly connected to social cognitive theory (SCT) [19,20,21,22]. SCT suggests that environment, behavior and individual cognition act together in individual activities [23,24]. Individuals are both shapers and products of the environment they are in. Based on SCT, Abubakar et al. [25] verified that safety behavior acts as a moderator when safety climate acts on work injuries, but not specifically on safety voice behavior. Shen et al. [26] found that construction workers were more willing to talk about safety-related matters in a good safety climate, but his study was based on social exchange theory. Few scholars have focused on the study of safety climate and safety voice from a social cognitive perspective.

Psychological safety is a feeling of individuals allowing them to express their opinions freely, without considering negative career consequences, self-image, or status. It is a perception that employees possess in communicating with their supervisors and colleagues, and is considered a critical factor that influences whether employees dare to express their ideas [27,28]. Pearsall and Ellis [29] argued that psychological safety facilitates employees to communicate openly, express suggestions or ideas, and seek feedback. Tucker and Turner [30] proposed that employee safety voice may be affected by the employees’ working environment, and verified that psychological safety takes a moderating role. In a good safety climate, employees can observe that others value safety, generate a sense of psychological safety, do not worry that expressing safety voice will bring them adverse consequences, and dare to express their concerns and opinions in safety [31]. Therefore, psychological safety can be a “bridge” between safety climate and safety voice. However, most current discussions on psychological safety and safety voice have been studied in a specific industry (e.g., health care workers (Hu and Casey [32]) and miners (Dodoo et al. [33])), and the findings lack generalizability [34]. In addition, most of the literature exploring psychological safety and safety voice is based on Western employees [30,32,33]. Due to cultural differences, the behaviors of Western and Chinese employees are very different [35]. Therefore, it is necessary to investigate the performance of Asian employees concerning psychological safety and safety voice. Given this, based on SCT, this paper aims to explore the impact of safety climate on safety voice and examine the mediating role of psychological safety. The study results will deepen and expand the research related to safety voice, which will help guide the management of enterprises and drive employee safety voice behavior, thus, promoting enterprises to improve their level of safety management.

## 2. Literature Review and Hypothesis

### 2.1. Safety Voice

Safety voice is the act of expressing perceived hazards in the workplace to avoid personal injury [36]. It can help organizations identify and eliminate safety hazards in time to avoid accidents, and many serious accidents have been attributed to a lack of safety voice [37,38]. Therefore, safety voice plays a critical role in maintaining organizational safety. Despite the significance of safety voice in an organization, employees face risks in expressing themselves. Employees are often concerned about the consequences of this risk and choose to hide their safety-related opinions. First, safety voice primarily targets the leaders. Safety voice often requires changes to existing systems and policies, which may be perceived as provocative, by the leaders. Leaders show aversion to employees’ asserting their authority through safety voice behavior, to prove themselves right [39]. Additionally, safety voice is more focused on the organization’s safety performance, with no significant increase in organizational productivity or economic benefits in the short term. Employees worry that safety voice will be seen by others as superfluous and detrimental to workplace survival.

Voice and safety voice are both ways for employees to express their opinions, however, safety voice differs from general employee voice. First, the focus of safety voice is different from that of general voice. Safety voice involves the disclosure of hazards in the workplace and concealing safety issues can lead to injury or death [18,40]; in contrast, general employee voice focuses on organizational decisions and operational practices [41]. In addition, safety voice intends to reduce accidents and, thus, keep employees safe [4], while general employee voice aims to improve organizational performance and help companies improve their economic performance [42].

Some scholars have studied safety voice as one dimension of safety citizenship behavior. Hofmann et al. [43] proposed the concept of safety organizational behavior, which is a voluntary individual behavior performed by employees to improve organizational performance. Hofmann et al. [43] proposed six components of organizational safety behavior: (i) helping, (ii) stewardship, (iii) initiating change, (iv) voice, (v) civic virtue, and (vi) whistleblowing. Curcuruto et al. [44] further divided safety citizenship behavior into proactive safety behavior and pro-social safety behavior based on Hofmann et al. [43], and regarded safety voice as proactive safety behavior. Many studies have adopted Curcuruto et al. [44]’s classification criteria [45,46,47,48].

In current research on safety voice, many scholars have used social exchange theory as the common ground theory [1,36,49,50]. Safety voice acts as an extra-role behavior, and organizations cannot force employees to express their safety opinions. In addition, employees may be perceived as “troublemakers” by their leaders and co-workers, causing interpersonal tensions [51]. Therefore, there is often a risk that employees will be pressured to remain silent. According to social exchange theory, good interaction between leaders and employees will form a social exchange relationship [52]. When leaders show concern and support for their employees, employees will take the initiative to implement extra-role behaviors. Therefore, supportive leadership behaviors (e.g., voice endorsement, Sun et al. [50]) motivate employee safety voice behavior. Turner et al. [3] investigated whether younger and adult employees behaved differently in terms of safety voice. They argued that according to age-related resource selectivity theory, when the supervisors’ safety commitment was unclear, adult workers were afraid of uncertainty factors, and their willingness to express safety voice was not as strong as that of younger employees. The results of subsequent empirical tests supported their hypothesis. Job demand–resources theory (JD-R) suggests that work demands consume human energy and cause adverse effects such as reduced productivity, while work resources provide support for employees and reduce their stress, thus, promoting them to be more engaged in their work [53]. Mathisen et al. [54] conducted a study based on JD-R and found that job demands inhibited safety voice, while job control promoted safety voice.

### 2.2. Safety Climate

The Israeli scholar Zohar [12] first put forward safety climate. It reflects the common perception of employees in an organization about a hazardous operating environment. Safety climate was later introduced into corporate safety practice. Safety climate has evolved and been generalized in various studies due to different research perspectives, supporting theories, and levels of analysis. It has been used, from time to time, interchangeably with the terms organizational climate and safety culture. Regarding safety climate, scholars currently have two definitions. The first defines safety climate as the perception of a safe work environment shared by all members within the organization [12,55]. The second definition proposes that safety climate can exist independently of the individual and is the objective environment [56]. This paper favors the first view, and defines safety climate as an overall assessment of the importance and implementation status of safety in the organization by all employees. Safety climate motivates employee safety behavior, reduces accident rates, and is essential for safety at work [6,57,58]. Employee behavior and safety climate are inextricably linked [59]. If the corporate safety climate is positive, employees sense that the environment is liberal and are more willing to talk openly about safety-related matters [60].

### 2.3. Psychological Safety

Psychological safety is a context where employees dare to show their true opinions in the organization without fear of such behavior affecting their career development and image [28]. According to Baer and Frese [61], psychological safety is an organizational, institutional procedure or norm that guides individuals within an organization to interact with each other in a trustful manner and effectively reduce the risks present in the corporate environment. The internal motivation of individuals and the process of shaping the psychological state of individual roles are influenced by psychological safety. When feeling enough psychological safety, employees show more work engagement [56]. Psychological safety contributes to organizational safety performance [62,63]. Newman et al. [64] argued that psychological safety is the employees’ judgment of their ability to freely express their opinions or views in the organization without fear. Research has shown that psychological safety can inhibit silent behavior, and can encourage employees to express their disagreements to superiors [65,66]. Many scholars have found that psychological safety can act as an intermediary in organizational practices and work behaviors [67,68,69].

### 2.4. Social Cognitive Theory

Bandura [23] first introduced the concept of SCT. SCT suggests that human learning behavior is generated by three aspects: human cognitive factors, environmental factors, and behavior, in which the person, environment, and behavior are interdependent and interact with each other. The theoretical model is characterized by three mechanisms: (1) the individual plays a dominant role in behavior, and the individual’s reflections from previous experiences will lead to the occurrence of the behavior, while the feedback from the results of behavior will also lead to self-regulation; (2) the environment as an external condition will determine the intensity of behavior, and at the same time, the individual will consciously change the environment to meet individual needs; and (3) the environment governs the formation of individual personality traits, while the individual’s cognition also acts on the surrounding environment. SCT breaks away from the single-factor decision theory of human behavior and proposes that individual behavior, individual cognition, and the external environment are viewed as a two-way interaction, e.g., individual and environmental interactions, i.e., the triadic interaction decision theory model.

It can be seen that individual behavior is determined by both an individual’s internal cognition and external environmental factors. Safety climate is the common risk perception formed by team members through safety training, safety management-related systems, and safety culture inculcation, which belongs to perceptible safety environment factors. Psychological safety is the perception that an employee’s image and career will not be negatively affected in the process of self-expression, and it belongs to individual cognitive factors. Safety voice is a constructive approach by employees towards making a difference to safety-related issues, which belongs to individual behavior. Safety climate and psychological safety interact to stimulate safety voice. Therefore, SCT is applicable to study the process of safety climate acting on safety voice. According to the triadic interaction model proposed by SCT, this paper follows the research path of “context-individual cognition-behavior”, treating safety climate as a contextual factor, and studies how it affects psychological safety and safety voice.

### 2.5. Hypothesis

#### 2.5.1. Safety Climate and Safety Voice

SCT provides unique insights for explaining the connection between safety climate and safety voice. According to SCT, safety voice is dependent on the contextual environment in which an individual lives. As safety voice is a dissatisfaction with, and threat to, the existing rules, processes, and systems, which can put pressure on other employees and threaten the interests of the old guard, the rejection of voice may be a way to balance team member relationships and maintain organizational stability, meaning that leaders may reject employee safety voice to maintain relationships. In addition, because speaking up about safety issues means challenging existing regulations, which may implicitly question a manager’s competence, managers may reject voice to maintain self-esteem and authority [70,71]. This resistance from leaders and co-workers can inhibit employees from expressing their opinions on safety. The safety climate will determine the employees’ understanding of the organization’s safe human factors environment, and employees will form judgments based on existing safety perceptions, systems, and procedures, which, in turn, will adjust their safety behaviors. Many scholars have found that antecedent variables of employee safety behaviors included safety climate [6,7,13,72]. Safety climate refers to the employees’ perception of the relative importance of workplace safety [12], and a high safety climate means that the company pays high attention to the safety environment and allocates various quality resources for safety, from the attitudes and behaviors of executives and colleagues to specific policies and procedures that emphasize providing sufficient guarantees for safety, and employees do not have to fear the adverse effects of safety voice. Mearns et al. [73] further confirmed that employees are more willing to communicate with the organization about safety when faced with a good climate, such as offering constructive comments and pointing out risks at work. Therefore, a hypothesis is proposed:

**Hypothesis** **1.** *Safety climate positively influences safety voice*.

#### 2.5.2. The Mediating Role of Psychological Safety

SCT suggests that individual behavior is not only related to the perception of the environment, but is also influenced by their psychological state [74]. Psychological safety expresses the employees’ internal evaluation of the organization’s environment, whereby employees judge the rationality of their forthcoming action. Safety climate can reflect the degree of organizational support for safety management, so organizations with a good safety climate attach importance to corporate safety management. At the same time, safety climate also emphasizes other safety management requirements, such as the identification of safety issues and, thus, avoidance of safety incidents [75]. Being in a clear, stable, and supportive work environment helps to enhance psychological safety. In a good organizational safety climate, employees who receive organizational attention and support for safety will gradually improve their psychological state at work and will not have much concern about engaging in safety behaviors that bring negative feedback. Antecedent studies of psychological safety have demonstrated that a safe climate is effective in increasing an individual’s psychological safety [76]. Therefore, we hypothesized that the safety climate can promote a sense of psychological safety:

**Hypothesis** **2.** *Safety climate positively influences psychological safety*.

Psychological safety is the sense that employees believe that they are expressing themselves in a way which will not cause harmful effects, and thereby damage to their image, status, and career. When individuals are psychologically safe, they tend to contribute ideas without fear of angering their leaders [77]. Safety voice behavior is a risky behavior because it is often accompanied by the discovery of problems and changes, which means that it may involve the destruction of interpersonal relationships among colleagues and challenges to the authority of superiors, so employees will always assess the risks behind the behavior and their ability to counteract them before expressing safety voice. Psychological safety expresses the evaluation of the risk outcomes to be considered in a particular situation (e.g., the workplace) [78]. When psychological safety is strong, employees do not have to worry that safety voice behavior will be ostracized and retaliated by colleagues, or that the safety voice behavior will be rejected by their superiors and, thus, affect their future development in the organization. Therefore, they tend to offer constructive ideas, express their views fully, and discuss existing problems in the organization [54]. Kark and Carmeli [79] noted that a sufficient sense of psychological safety can facilitate employees’ expression of ideas on the job. When psychological safety is weak, employees are more inclined to be silent [80].

SCT points out that the external environment not only acts directly on individual behavior but also affects behavior through internal factors of the individual [81]. Therefore, we believe that the safety climate as an external environmental factor can not only directly promote employees to express safety voice behavior, but also motivate employee safety voice behavior through their psychological factors, namely, psychological safety. Safety voice is a challenging behavior that leads employees to be “labeled” as bad, and possibly even chastised. Psychological safety, as a positive perception of whether an individual’s behavior is “safe,” reduces employees’ risk perception of presenting new ideas, and motivates employees to speak up [82]. Therefore, we make the hypotheses:

**Hypothesis** **3.** *Psychological safety positively influences safety voice*.

**Hypothesis** **4.** *Psychological safety plays a mediating role in safety climate and employee safety voice*.

**Figure 1:**
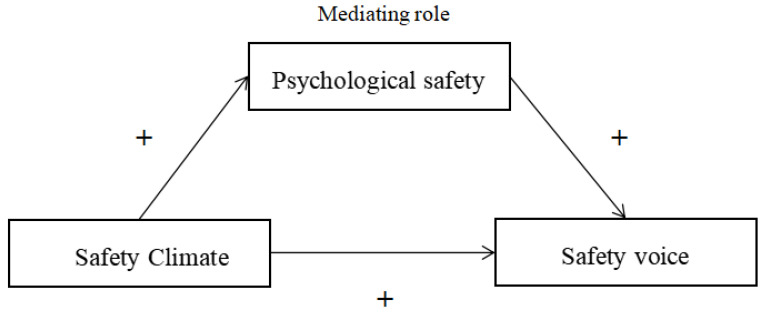
Research hypothesis model.

From a social cognitive perspective, we developed a research hypothesis model (as shown in Figure 1) to examine how the safety climate affects safety voice, and the role of psychological safety between them. In detail, the model is used to explore the positive relationship between safety climate and safety voice, and whether psychological safety plays a positive mediating role.

## 3. Methods

### 3.1. Sample and Procedure

The research sample was drawn from employees of three companies in Sichuan, Jiangxi, and Chongqing, mainly in manufacturing and construction industries. Collecting data from different industries can improve the generalizability of the study results [34]. We distributed 270 questionnaires. After excluding the unqualified questionnaires that were missed and those with more concentrated answers, 249 valid questionnaires were collected. The return rate of the questionnaires was 92.2%. Table 1 shows the information of the valid sample.

The questionnaire collection for this study lasted about two months from beginning to end, and the data were collected using paper questionnaires. The research team was divided into three groups to visit the three different companies and distribute the paper questionnaires. The study description was attached to the questionnaires, emphasizing that the questionnaires were anonymous and that the study was completely voluntary, eliminating any worries of the employees.

### 3.2. Variable Measurement

In addition to the title and greeting, the research questionnaire consisted of the safety climate scale, the psychological safety scale, and the safety voice scale. We chose mature scales, and due to differences in language habits and expressions, the back-translation method was used. First, we translated the scales into Chinese, and then asked three native Chinese speakers who were proficient in English to back-translate the Chinese questionnaire into English. Then we compared it with the original questionnaire, focusing on the modified, significantly different items, and back-translated them again, thus, determining the scale items of the formal questionnaire. The Likert 5-point scale was used for all questions (1 for totally agree, 5 for totally disagree), which finally formed the research questionnaire. We showed the measured items in Appendix A.

#### 3.2.1. Safety Climate

The developed scale was used to measure safety climate. The scale included three questions. Typical items such as “Safety is given a high priority by management” and “Management considers safety to be important” were completed by employees. In this research, the Alpha coefficient for safety climate was 0.794.

#### 3.2.2. Safety Voice

In this research, safety voice was measured using the scale designed by Tucker et al. [36], which contained five items, which were typically measuring questions such as “I make suggestions about how safety can be improved”. Herachwati et al. [14] used this scale and demonstrated its good reliability and validity. The Alpha coefficient of safety voice was 0.735.

#### 3.2.3. Psychological Safety

The one-dimensional scale of psychological safety by Liang et al. [82] was used, which was composed of five items, including typical items such as “In my work unit, I can express my true feelings regarding my job” and “In my work unit, I can freely express my thoughts”. It was completed by employees. The Alpha coefficient of psychological safety was 0.813.

#### 3.2.4. Control Variables

We chose gender, age, education, position, and working years as the control variables. Turner et al. [3] verified that younger employees were significantly more willing to become involved in safety voice than older employees, when the safety commitment of their supervisors was unclear. Tucker and Turner [19] argued that employees’ pointing out safety issues was related to gender, with female employees being more willing to engage in safety voice compared with males. In addition, education, position, and working years can also influence employees’ expression of work-related ideas [83,84]. Therefore, in this study we used them as control variables.

## 4. Results

### 4.1. Validity Analysis

Using Mplus 8.3 software, the variables were subjected to CFA and, thus, we obtained discriminant validity. We used mature scales to measure the variables, and the dimensional structure of the variables was determined. CFA was used to test whether the data obtained from the study could be fitted well with the dimensional structure of the variables set in advance [85]. CFA requires certain fit indicators to determine whether the model is stable. Among the commonly used fit indicators, CFI and TLI should not be lower than 0.9, the value of $X^{2}/df$ should be less than 3, and RMSEA should not exceed 0.08 [86,87]. We compared the hypothetical three-factor model with four alternative models. As seen in Table 2, four alternative models did not meet the structural validity requirements. The three-factor model, including safety climate, psychological safety, and safety voice, had good structural validity: $X^{2}/df$ equaled 2.268, which is less than 3; RMSEA equaled 0.071, which is less than 0.08; CFI equaled 0.930, which is greater than 0.9; TLI equaled 0.911, which is greater than 0.9. In summary, the fit indices of the three-factor model were better than the other model. All were within the acceptable range, which indicated that the model structure was reasonable.

### 4.2. Homogeneous Bias Analysis

Given that the questionnaires were filled by employees, the subjective factors of employees may have led to homogeneous bias in the measured variables. We used SPSS 26.0 to conduct Harman’s one-way test method to perform exploratory factor analysis (EFA) for all variables. Podsakoff et al. [88] considered a one-way cumulative variance interpretation rate of 40% to be acceptable. We extracted three factors from the scales, and the first factor extracted explained 37.097% of the variance, i.e., not exceeding 40%. Therefore, there was no single factor that could explain most of the variance in this study, i.e., the problem of homogeneous bias in the questionnaire data was not significant, and did not have a serious impact on the scales.

### 4.3. Descriptive Analysis

The results of the descriptive statistics are shown in Table 3. There was a correlation coefficient of 0.406 between safety climate and psychological safety, and the significance level was *p* < 0.01, which indicates that they were positively associated. The correlation coefficient between safety climate and employee safety voice was 0.524, and the significance level was *p* < 0.01, meaning that safety climate and employee safety voice had a positive correlation. Psychological safety and safety voice had a positive correlation, with the correlation coefficient being 0.444 and the significance level being *p* < 0.01. The above results supported the hypotheses.

### 4.4. Hypothesis Test

#### 4.4.1. Main Effects Test

As shown in Table 4, hierarchical regression analysis was used to test the main effects. Model 1 was a regression analysis in which gender, age, education, position, and working years were independent variables, and psychological safety was the dependent variable. Model 2 was a regression analysis based on model 1. In model 2, we added safety climate. The $R^{2}$ of model 1 was 0.046, indicating that gender, age, education, position, and working years explained only 4.6% of the variation in the causes of safety voice. Relative to the control variables, the variable safety climate increased explanatory power on psychological safety with an increase in ∆$R^{2}$ of 0.137, indicating that the addition of safety climate enhanced the explanatory power by 13.7%. The coefficient of influence of safety climate as an independent variable on psychological safety was 0.385 and showed significance (*p* = 0.000 < 0.01). Therefore, safety climate can promote psychological safety, and the second hypothesis held.

In Model 3, the control variables were the independent variables and safety voice was the dependent variable. Model 4 was a regression analysis based on model 3 with safety climate as the independent variable. The $R^{2}$ of model 3 was 0.145, indicating that gender, age, position, education level, and working years explained only 14.5% of the variation in safety voice. Relative to the control variables, the introduction of the variable safety climate had increased explanatory power on employee safety voice behavior with an increase in ∆$R^{2}$ of 0.197, indicating that the addition of safety climate enhanced the explanatory power by 19.7%. The coefficient of influence of safety climate as an independent variable on safety voice was 0.461 and showed significance (*p* = 0.000 < 0.01). Thus, safety climate positively acts on safety voice, and the first hypothesis was supported.

We added psychological safety as an independent variable to model 3 to form model 5. Model 3 had an ∆$R^{2}$ of 0.145, and relative to the control variables, the introduction of safety climate increased ∆$R^{2}$ by 0.145, indicating that the addition of safety climate enhanced the explanatory power by 14.5%, and psychological safety positively affected safety voice (β = 0.390, *p* < 0.01), supporting the third hypothesis.

#### 4.4.2. Mediating Effect Test

To test the role of psychological safety, we used the stepwise regression test and bootstrap sampling method, respectively.

(1) Stepwise regression test

In a stepwise regression test, when the addition of a variable causes a change in the correlation coefficient between the independent and dependent variables, it plays a mediating role. According to Table 5, there was a direct relationship between safety climate and safety voice (β = 0.461, *p* < 0.001). When psychological safety was introduced, the link was still positive, but the decrease in the regression coefficient (β = 0.363, *p* < 0.001) indicated that psychological safety functioned as a clear intermediary.

(2) Bootstrap sampling method

We again tested the interval estimation method using bootstrap sampling. The bootstrap sampling method calculates the confidence interval by sampling the sample a certain number of times (usually not less than 1000) with put-back repetitions, and if the confidence interval does not contain 0, the mediation effect is valid [89]. We used the Process plug-in to select a sample size of 1000 and to test the 95% confidence interval. In this case, safety voice was used as the dependent variable, safety climate as the independent variable, and psychological safety as the mediating variable.

Table 6 shows the test results. The results indicated that 0 (95% CI: 0.0516–0.1793) was not in the 95% confidence interval, and the path was significant. Therefore, safety climate acts indirectly on safety voice through psychological safety. The indirect effect value of safety climate on safety voice through the mediation of psychological safety was 0.1056, so the fourth hypothesis is valid.

## 5. Discussion

### 5.1. Discussion of Results

From the analysis, it was clear that safety voice was affected positively by the safety climate (β = 0.461, *p* < 0.01). In the literature on employee safety voice, environmental and individual factors are considered to be the main factors influencing employee behavior [18,90,91]. Safety climate is a common risk perception developed by team members through safety training, safety management-related systems, and safety culture inculcation [55]. To some extent, safety climate may explain the perception, support, or policy-making gap, between management and employees [92]. Safety climate can motivate employees to become involved in safety [75,76]. Employee-perceived safety support has been shown to play a moderating role between organizational safety openness and employee safety voice [25]. Curcuruto et al. [93] found that supervisors’ and colleagues’ safety support facilitated safety voice. Thus, safety climate can promote more safety voice behavior by reducing employees’ psychological burden.

In addition, we found safety climate improved psychological safety (β = 0.385, *p* < 0.01). SCT suggests the situational stimulation will impact individual behavioral responses by acting on individual cognition [81]. Before employees express their opinions, they evaluate their surroundings. A positive safety climate means that the enterprise takes safety seriously, i.e., a higher level of attention to safety and stronger support for safety management. Employees feel greater psychological safety in a relatively supportive environment [94], so a positive organizational safety climate is conducive to psychological safety.

According to the results, psychological safety was positively associated with safety voice (β = 0.390, *p* < 0.01). By testing the mediating effect, we found that safety climate can positively influence safety voice through psychological safety. SCT proposes that an individual’s specific behavior depends on three factors: environment, individual, and behavior, where external contextual factors influence individual behavior through the “context-subject perception-behavior” pathway [95]. The employees’ safety perceptions go through a psychological decision-making process. On the one hand, when employees face a work environment that ignores safety, employees’ psychological safety is low, due to the pressure of unemployment risk. They tend to choose avoidance behavior due to “interpersonal risk behavior.” However, when the organizational safety climate is high, employees face less pressure from interpersonal risks or other negative effects, their psychological safety is at a higher level, and they prefer to speak out about safety hazards. On the other hand, psychological safety can act directly on safety voice. Psychological safety facilitates employees to express suggestions and seek feedback [78]. Higher psychological safety facilitates employees’ level of engagement and involvement at work, which leads to more exploratory and innovative behaviors [96]. When employees’ psychological safety is high, employees are less likely to worry about interpersonal risks and are more likely to offer constructive ideas [64]. Safety-constructive comments are full of uncertainty and risk, which may bring certain risks and costs to employees, making them afraid to easily initiate safety constructive behaviors. Employees hold higher safety beliefs that they can freely express their ideas when their psychological safety is relatively high; therefore, higher levels of psychological safety can motivate and promote safety voice behavior.

### 5.2. Research Implications

#### 5.2.1. Theoretical Implications

First, this study deepened the study of safety voice boundary conditions from a work context perspective. Much previous research has paid attention to the impact of safety climate on employees’ safety behavior, but a direct exploration of safety voice behavior has been lacking [6,7,72]. When employees perceive a strong safety climate, they dare to become involved in safety efforts, such as wearing safety gear, complying with various safety regulations, and expressing safety-related comments [97,98,99]. Based on SCT, this paper regards safety climate as a situational factor to convey the high priority of safety to employees [8]. Safety climate then acts on psychological safety to finally stimulate employee safety voice, expanding the research on the antecedent variables of safety voice.

Second, this paper built a model that explored the mediating effect of psychological safety. Both trust and leadership openness affect employees’ expression of opinions at work, through psychological safety [27,30]. A good safety climate will increase employees’ psychological safety. Employees will not be overly concerned about their words and actions negatively impacting their workplace survival after they feel the importance of safety to the organization and its support for safe work, and their willingness to participate and comply with safety behaviors will increase. Hence, their willingness to express safety voice behavior is higher. The mediating model provides an idea for the boundary conditions of safety climate affecting safety voice.

Finally, we examined the mechanism of how safety climate acts on safety voice from a social cognitive perspective, providing a new perspective for research on safety climate influence on employees’ safety-related behaviors. Many scholars have studied safety voice from the perspective of social exchange theory. Employees implement safety voice behavior to reciprocate the support of the organization and improve the organization’s safety performance [14,49]. Unlike the explanatory mechanism of social exchange theory, this paper adopted the triadic interaction model proposed by SCT, which follows the “context-individual cognition-behavior” path [23,100] and considers safety climate as a contextual factor. The triadic interaction model goes beyond the traditional psychological “one-way determinism” and integrates the individual, the environment, and the behavior, and builds a bridge between the individual perception and situations around the behavior. This paper revealed the function of safety climate in facilitating safety voice, which enriches the application of SCT in the study of employee safety behavior. It provides an essential reference for the formation mechanism of safety voice.

#### 5.2.2. Practical Implications

First, the research results show that safety climate can positively predict safety voice behavior. In a workplace where safety is prized, employees dare to speak up about safety matters. Therefore, corporate management should strive to create a good safety climate through the setting of reasonable regulations and daily practices. In terms of rules and regulations, leaders can establish a reward system that awards bonuses or promotions to employees who propose safety suggestions that are adopted. In daily practice, leaders should often talk to employees about safety matters and encourage them to speak up. They can also organize seminars and invite front-line employees to focus on the shortcomings of work safety and ways to improve it.

In addition, psychological safety can facilitate employees talking about safety. Therefore, in daily management, enterprises should take the construction of employees’ psychological safety seriously, to enhance psychological safety and eliminate employees’ worries about the negative consequences of safety voice. On the one hand, managers should maintain a humble attitude and actively accept the opinions of employees. When the opinions put forward by employees receive positive feedback from their leaders, they will put aside their fear of leadership authority and be brave enough to put forward more safety ideas [50]. On the other hand, leaders should be aware of the psychological state of employees and communicate with them in a timely manner. If leaders find that employees have a negative state of mind such as burnout and depression, they should give employees material or moral support. When employees feel the organization’s care, their psychological safety will also be improved and they will bravely make comments [27].

### 5.3. Limitations

This study has some shortcomings. First, the questionnaire data collected in this study were all obtained from employee self-assessments. Although some scholars have found that safety climate and psychological safety can act on work behavior through employee self-assessment [6,101], employee self-assessments may lead to the generation of homogeneous errors. Although this paper used the homogeneous analysis of variance to rule out the severity of common method bias, its effect cannot be eliminated from a statistical perspective alone. In subsequent study, the measurement accuracy could be improved by collating information from different sources through multiple source assessments (e.g., supervisors, family, and co-workers).

Second, the study only examined safety climate acting on safety voice through psychological safety; there may be other paths, as well as boundary conditions, that were not included in this study. Future research should introduce different variables from multiple perspectives to discuss how safety climate works for safety voice.

Third, the research data came from construction and manufacturing enterprises in Sichuan, Jiangxi, and Chongqing, and data from employees in other types of enterprises and regions could not be obtained. Difference in occupation is also an essential factor leading to changes in employees’ willingness to further express safety voice behavior [14]. Future studies should increase the sample size to further validate the conclusions, and data should be collected from employees in other types of companies to explore the generality of the results.

Fourth, in our sample, people under the age of 25 accounted for the largest proportion of respondents, at 38.2%, and more than half (55.0%) of employees had less than three years of work experience. Turner et al. [3] found that younger and older employees exhibited different safety voice behaviors due to different work experiences; when leaders did not make visible safety commitments, older employees were more reluctant to voice their opinions [3]. Therefore, future studies should increase the sample to include more people of older age and more years of working experience, to explore whether new conclusions will emerge.

Finally, in terms of study design, on the one hand we used a cross-sectional study that could only verify the contribution of safety climate to safety voice. According to SCT, environment and individual behavior influence and interact with each other [23]. Employees are willing to voice safety concerns in an atmosphere where everyone values safety, and, in turn, employees’ frequent voicing of safety ideas may contribute to a good safety climate. Therefore, future studies could use a longitudinal design to explore whether safety voice and safety climate interact. On the other hand, we used a safety climate scale that only dealt with management’s attitude toward safety. Many current related studies have divided safety climate into several dimensions, so, in the future, we could use a safety climate scale with more dimensions to make the study more rigorous.

## 6. Conclusions

Based on the ternary interaction model of SCT, this study organically linked safety climate, safety voice, and psychological safety. It investigated the influence of safety climate on safety voice and the role of psychological safety in between. We found that safety climate positively affected safety voice behavior, and that psychological safety mediated their relationship. With the complex changes and rapid development of modern enterprises, motivating and improving safety voice behavior is crucial to the long-term development of enterprises. These results can help to further stimulate and enhance employee safety voice; they also have essential reference values for improving corporate safety decisions and optimizing safety management systems.

## Figures and Tables

**Table 1:** Sample basic information.

Items	Sample Classification	Number	Percentage (%)
Gender	Male	139	55.8
	Female	110	44.2
Age	25 years old and below	95	38.2
	26–35 years old	85	34.1
	36–45 years old	53	21.3
	45 years old and above	16	6.4
Education Level	High school and below	19	7.6
	College	50	20.1
	Bachelor	122	49.0
	Postgraduate and above	58	23.3
Position	Front-line employee	164	65.9
	Grass-roots manager	58	23.3
	Middle/Senior management	27	10.8
Work Experience	1 year and below	87	34.9
	1–3 years	50	20.1
	3–7 years	74	29.7
	7 years and above	38	15.3

**Table 2:** Results of confirmatory factor analysis (N = 249).

Model	Factor	$\;\;X^{2}$	df	$X^{2}/df$	RMSEA	CFI	TLI
Three-factor model	SC, PS, SV	138.363	61	2.268	0.071	0.930	0.911
Two-factor model A	SC and PS, SV	337.516	64	5.274	0.131	0.753	0.699
Two-factor model B	SC, PS and SV	293.465	64	4.585	0.120	0.793	0.747
Two-factor model C	SC and SV, PS	262.962	64	4.109	0.112	0.820	0.781
One-factor model	SC, PS and SV	409.704	65	6.303	0.146	0.689	0.626

Notes: SC = safety climate, PS = psychological safety, SV = safety voice.

**Table 3:** Results of descriptive analysis.

Variable	M	SD	1	2	3	4	5	6	7	8
1. Gender	1.44	0.5	1							
2. Age	1.96	0.92	−0.338 **	1						
3. Education	2.88	0.85	0.78	−0.185 **	1					
4. Position	1.45	0.68	−0.112	0.406 **	0.287 **	1				
5. Working years	2.25	1.09	−0.413 **	0.752 **	−0.067	0.451 **	1			
6. Safety climate	4.02	0.63	−0.033	−0.033	0.185 **	0.227 **	0.12	1		
7. Psychological safety	3.39	0.7	−0.079	0.106	0.054	0.185 **	0.167 **	0.406 **	1	
8. Safety voice	3.75	0.6	−0.063 **	0.169 **	0.224 **	0.344 **	0.212 **	0.524 **	0.444 **	1

Notes: ** *p* < 0.01; 1 = male.

**Table 4:** Results of the main effects test.

Variable/ Model	Psychological Safety		Safety Voice		
	Model 1	Model 2	Model 3	Model 4	Model 5
Gender	−0.028	−0.025	−0.001	0.002	0.010
Age	−0.063	−0.016	0.037	0.094	0.062
Education	0.014	−0.032	0.168 *	0.112	0.162 **
Position	0.140	0.071	0.242 **	0.159 *	0.187 **
Working years	0.140	0.088	0.086	0.023	0.031
Safety climate		0.385 ***		0.461 ***	
Psychological safety					0.390 ***
F	0.323 *	9.013 ***	8.260 ***	20.970 ***	16.503 ***
$R^{2}$	0.046	0.183	0.145	0.342	0.290
$\Delta R^{2}$		0.137		0.197	0.145

Notes: * *p* < 0.05; ** *p* < 0.01; *** *p* < 0.001.

**Table 5:** Results of stepwise regression analysis.

Variable/Model	Safety Voice	
	Model 6	Model 7
Gender	0.002	0.009
Age	0.094	0.098
Education	0.112	0.120 *
Position	0.159 *	0.141 *
Working years	0.023	0.000
Safety climate	0.461 ***	0.363 ***
Psychological safety		0.254 ***
F	20.970 ***	22.473 ***
$R^{2}$	0.342	0.395
$\Delta R^{2}$		0.053

Notes: * *p* < 0.05; *** *p* < 0.001.

**Table 6:** Results of the bootstrap sampling method.

	Effect	Boot SE	Boot LLCI	Boot ULCI	Relative Effect Value
Total effect	0.4914	0.0508	0.3913	0.5915	
Direct effect	0.3858	0.5320	0.2810	0.4906	78.51%
Mediating effect	0.1056	0.0333	0.0516	0.1793	21.49%

## Data Availability

The data presented in this study are available on request from the corresponding author.

## Appendix A

**Table A1:** Scale items.

Variables	Author	Items
Safety Climate	Neal et al. [102]	1. Management places a strong emphasis on workplace health and safety. 2. Safety is given a high priority by management. 3. Management considers safety to be important.
Psychological Safety	Liang et al. [82]	1. In my work unit, I can express my true feelings regarding my job. 2. In my work unit, I can freely express my thoughts. 3. In my work unit, expressing your true feelings is welcomed. 4. Nobody in my unit will pick on me even if I have different opinions. 5. I’m worried that expressing true thoughts in my workplace would do harm to myself (reverse-coded).
Safety Voice	Tucker et al. [36]	1. I make suggestions about how safety can be improved. 2. I tell my colleague who is doing something unsafe to stop. 3. I discuss new ways to improve safe driving with my colleagues or boss. 4. I inform the union/boss when I notice a potential driving hazard. 5. I report to my boss if my colleagues break any safety rules.
